# Sudden Ventilatory Failure Caused by Mucus Plugging of the Endotracheal Tube in a Neonate With Congenital Pneumonia During Oral Surgery: A Case Report

**DOI:** 10.7759/cureus.109336

**Published:** 2026-05-21

**Authors:** Eunkyung Choi, Juhee Min, Deokhee Lee, Hyunho Shin

**Affiliations:** 1 Department of Anesthesiology and Pain Medicine, Yeungnam University College of Medicine, Daegu, KOR

**Keywords:** airway management, congenital pneumonia, endotracheal tube obstruction, general anesthesia, mucus plugging, neonate

## Abstract

Endotracheal tube (ETT) obstruction is a rare but potentially life-threatening complication of general anesthesia, particularly in neonates with increased airway secretions. We report a case involving a 22-day-old neonate with congenital pneumonia who developed sudden ventilatory failure during oral surgery under general anesthesia. Abrupt increases in airway pressure and reduced tidal volume were observed, and suctioning was unsuccessful. ETT exchange immediately restored ventilation, and the removed tube was found to be obstructed by viscous mucus. This case highlights that acute ETT obstruction can occur unexpectedly, even shortly after intubation. Early recognition and prompt airway intervention are essential to prevent severe complications.

## Introduction

Managing neonatal airways during general anesthesia is uniquely challenging due to anatomical and physiological vulnerabilities, including small airway caliber and limited respiratory reserve [1,2]. These factors render neonates particularly susceptible to rapid deterioration even with minor airway compromise. Among perioperative complications, endotracheal tube (ETT) obstruction is potentially life-threatening and may cause abruptly increased airway pressure, reduced tidal volume, and diminished breath sounds.

Congenital pneumonia further increases the risk of airway obstruction by promoting excessive and viscous airway secretions. In such patients, mucus accumulation within a narrow ETT can rapidly lead to critical ventilatory impairment. However, acute ETT obstruction may occur unexpectedly even shortly after intubation and may present similarly to other causes of intraoperative ventilatory difficulty, such as bronchospasm, tube malposition, or equipment malfunction, potentially delaying appropriate management [3].

Here, we report a case of sudden ventilatory failure caused by mucus plugging of the ETT during oral surgery in a neonate with a congenital pneumonia history. This case highlights the importance of early recognition and prompt differentiation of acute ETT obstruction from other causes of increased airway pressure in vulnerable pediatric patients, even shortly after intubation.

## Case presentation

A 22-day-old female neonate weighing 3,090 g (gestational age of 38 weeks and 4 days, birth weight 2,900 g) was diagnosed with a 0.5 x 0.5 cm cystic mass at the base of the tongue and scheduled for surgical excision. Her medical history was notable for congenital pneumonia diagnosed on the second day of life, for which she received supportive care and antibiotic therapy. The surgery was performed 20 days after her pneumonia diagnosis because the cystic tongue-base lesion posed a potential risk of airway compromise and feeding difficulty. Although her respiratory symptoms had gradually improved, with oxygen saturation maintained at 98-99% on room air, mild chest retraction and stridor persisted.

**Figure 1:**
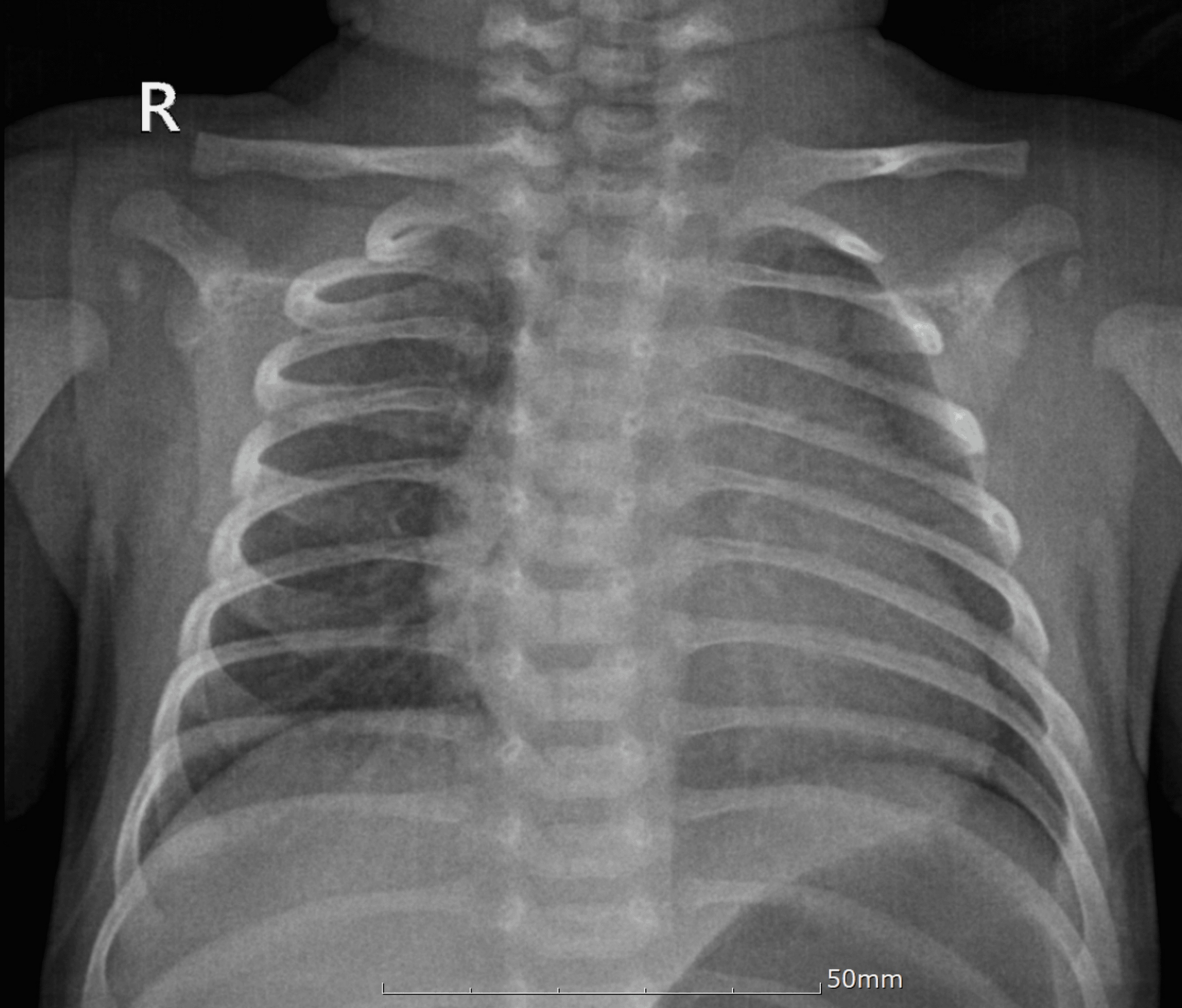
Chest radiography demonstrated bilateral lower lung field consolidations. The cardiac silhouette is within normal limits, and the bilateral costophrenic angles are preserved without evidence of pleural effusion.

On the day of surgery, the patient’s oxygen saturation on room air was 98-99%. Chest radiography demonstrated bilateral lower lung field consolidations (Figure 1). Laboratory findings were within normal limits (Table 1). 

**Table 1:** Routine laboratory tests were within the normal range.

Parameter	Result	Reference range	Unit
White blood cells	12.21	9-30	×10³/µL
Hemoglobin	11.4	10-16	g/dL
Platelet	461	150-600	×10³/µL
Sodium	137	135-145	mEq/L
Potassium	5.2	3.5-5.5	mEq/L
Chloride	106	98-110	mEq/L
AST	35	9-80	U/L
ALT	14	0-40	U/L
BUN	4.4	3-12	mg/dL
Creatinine	0.33	0.2-0.5	mg/dL
TSH	3.74	1-10	µIU/mL
Free T4	1.65	1-2.5	ng/dL

**Figure 2:**
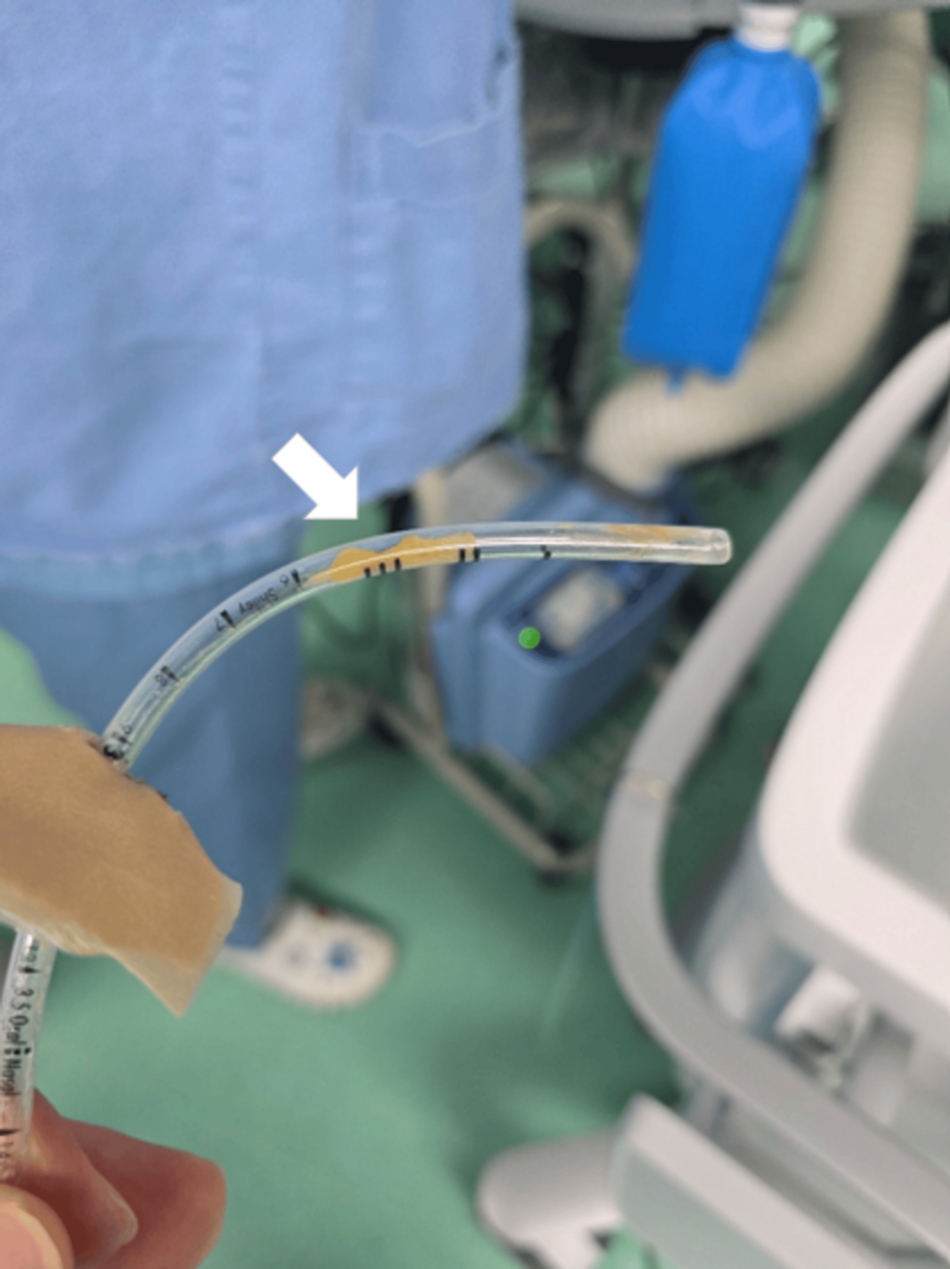
Tube partially occluded by yellow mucoid secretion (white arrow).

No premedication was administered, and general anesthesia was induced with thiopental and rocuronium. After achieving adequate mask ventilation, endotracheal intubation was performed using direct laryngoscopy with a 3.0 mm cuffed ETT (Shiley™, Covidien, USA). Intubation was successful on the first attempt without significant difficulty. A considerable amount of secretion was noted during endotracheal and oral suctioning. Anesthetic maintenance was achieved with sevoflurane 1.7% by volume in 50% FiO2 at a total fresh gas flow of 3 L/min. The ventilator was set to volume-guaranteed pressure mode with a tidal volume of 27 mL, a respiratory rate of 40 breaths per minute, and a positive end-expiratory pressure (PEEP) of 4 cmH₂O, resulting in a peak inspiratory pressure (PIP) of 17 cmH₂O. After induction, vital signs included blood pressure (BP) 89/45 mmHg, heart rate (HR) 160 bpm, SpO2 100%, and end-tidal CO2 30 mmHg. Thirty minutes after induction, we observed signs of partial endotracheal obstruction, including a sudden increase in PIP to over 30 cmH₂O, decreased tidal volume below 20 mL, and transient capnography waveform reduction followed by EtCO₂ elevation above 45 mmHg. Ventilation was switched from mechanical to manual, and FiO₂ was increased to 100%. However, manual ventilation remained difficult, with high resistance to bagging and inadequate tidal volume. Oxygen saturation remained between 96 and 100% during the event. The anesthesia circuit, ventilator system, tube depth, and possible tube kinking were immediately assessed and found to be unremarkable. Bilaterally decreased breath sounds without wheezing were noted, and the surgeon identified secretions within the endotracheal tube during laryngoscopy. Passage of a 6-Fr suction catheter was unsuccessful because highly viscous mucus at the distal lumen markedly limited catheter advancement, resulting in ineffective suctioning despite partial luminal patency. Pneumothorax was considered less likely because ventilation rapidly normalized after ETT exchange. The ETT was exchanged for a new 3.0 mm cuffed tube (Shiley™, Covidien, USA). The removed tube revealed partial distal luminal obstruction caused by thick yellow mucoid secretion. Following tube exchange, PIP decreased to baseline levels, tidal volume normalized, and capnography waveform recovered immediately without further ventilatory difficulty. We changed the ETT to a new 3.0 mm tube (Shiley™, Covidien, USA). The removed tube revealed partial distal lumen obstruction due to yellow mucoid secretion (Figure 2).

Following tube exchange, ventilation normalized, and there were no further ventilation issues. The patient remained hemodynamically stable, and oxygen saturation by pulse oximetry was maintained at 96-100% throughout the procedure. Fifteen minutes later, neuromuscular blockade was reversed with pyridostigmine 0.2 mg/kg and glycopyrrolate 0.01 mg/kg. The patient was discharged on postoperative day six without any notable complications.

## Discussion

Airway management in neonates during general anesthesia remains particularly demanding, given their distinct airway anatomy and limited respiratory reserve. Compared to older children, neonates have a relatively large tongue, a more cephalad larynx, and narrow, compliant airways, which increase their susceptibility to rapid airway obstruction [2]. Additionally, high oxygen consumption and limited functional residual capacity result in rapid desaturation during even brief periods of hypoventilation or apnea [1]. These factors, combined with immature airway reflexes and increased vulnerability to airway edema, contribute to a heightened risk of perioperative respiratory complications [4]. Such vulnerabilities are further exacerbated during oral cavity procedures, where surgical manipulation may compromise ventilation. In the present case, although mild respiratory symptoms persisted, the risks and benefits of delaying surgery versus proceeding under controlled airway management conditions were carefully considered because the tongue-base lesion carried a potential risk of airway compromise and feeding difficulty.

Several perioperative factors likely contributed to airway obstruction development. Congenital pneumonia is associated with increased airway secretions and higher mucus viscosity, predisposing patients to tenacious plug formation [5,6]. Previous reports have described airway obstruction caused by viscous secretions in patients with underlying respiratory conditions, further supporting this pathophysiological mechanism [7,8]. Children with recent or ongoing respiratory infections are at increased risk for perioperative respiratory complications, particularly in the presence of copious airway secretions [9]. During general anesthesia, mucociliary clearance is reduced, and the cough reflex is suppressed, promoting airway secretion retention [10]. Additionally, perioperative factors such as dry inspired gases and the effects of anesthetic agents may increase mucus viscosity and further impair mucous transport [11]. In the present case, active humidification was not routinely applied during the relatively short procedure, which may have contributed to secretion thickening. In neonates with copious airway secretions or recent respiratory infection, use of a heat and moisture exchanger or active humidification system may help reduce secretion thickening and the risk of ETT obstruction. Finally, the shared airway during oral cavity surgery may delay recognition and removal of obstructive material.

Although ETT obstruction is more common after prolonged ventilation, it can also occur shortly after intubation due to highly viscous secretions. Airway obstruction during general anesthesia typically presents with a sudden increase in peak airway pressure, decreased tidal volume, and diminished or absent breath sounds. Capnography may reveal reduced or absent end-tidal CO₂, depending on obstruction severity. However, these findings are not specific and can overlap with other causes of intraoperative ventilatory difficulty, including bronchospasm, ETT kinking or malposition, and equipment malfunction [12]. Therefore, rapid and systematic evaluation of the anesthesia circuit, ventilator system, tube position and patency, and pulmonary complications is essential for identifying the underlying cause and initiating appropriate management. Notably, bronchospasm is typically associated with wheezing, a prolonged expiratory phase, and partial response to bronchodilators, whereas mechanical obstruction often shows minimal improvement despite airway pressure support [13]. It is more commonly observed during light anesthesia, particularly during induction or emergence, when airway reflexes are more easily stimulated. In contrast, mechanical obstruction, such as mucus plugging and foreign bodies, often leads to more abrupt deterioration with minimal improvement following bronchodilator therapy [2].

When ETT obstruction is suspected, suctioning and bronchoscopic evaluation are generally recommended [10]. However, in neonates, these approaches may be limited by the small airway diameter. In such situations, prompt ETT exchange may be the most effective intervention. However, during oral surgery, this may be particularly challenging because blood and secretions can obscure the airway and increase the risks of tube exchange. Although neonatal flexible bronchoscopes are available, they may be restricted by the ETT’s small internal diameter, potential ventilation impairment during the procedure, and limited resources in some clinical settings. Thus, prompt tube exchange can be critical in emergencies. In this case, immediate tube exchange resulted in rapid ventilation restoration, supporting the diagnosis of ETT obstruction due to mucus plugging and highlighting the importance of early recognition and decisive airway management. This case underscores the importance of maintaining a high index of suspicion for airway obstruction in neonates, particularly those with pre-existing pulmonary conditions. Preventive strategies such as adequate humidification, frequent airway suctioning, and careful monitoring of ventilatory parameters are essential. Finally, clinicians should be prepared for rapid airway intervention when sudden ventilatory compromise occurs, especially in shared airway procedures where access may be limited.

## Conclusions

This case illustrates that ETT obstruction caused by highly viscous secretions can develop abruptly in neonates, even shortly after intubation. Intraoperative ventilatory deterioration in this population may present with nonspecific findings, making it difficult to distinguish from bronchospasm or equipment-related issues. Therefore, clinicians should remain alert to the possibility of mechanical obstruction, particularly in patients with recent pulmonary pathology and during shared airway procedures. When conventional measures such as suctioning fail or are not feasible, early consideration of ETT replacement may be crucial for restoring effective ventilation. Proactive strategies, including optimization of airway humidification and close monitoring of ventilatory trends, may help reduce the risk of similar events.
